# Resistance to Cereal Cyst Nematodes in Wheat and Barley: An Emphasis on Classical and Modern Approaches

**DOI:** 10.3390/ijms20020432

**Published:** 2019-01-19

**Authors:** Muhammad Amjad Ali, Mahpara Shahzadi, Adil Zahoor, Abdelfattah A. Dababat, Halil Toktay, Allah Bakhsh, Muhammad Azher Nawaz, Hongjie Li

**Affiliations:** 1 Department of Plant Pathology, Faculty of Agriculture, University of Agriculture, Faisalabad 38040, Pakistan; mahpara015@gmail.com (M.S.); adilzahoor3253@gmail.com (A.Z.); 2 International Maize and Wheat Improvement Center (CIMMYT), Ankara 06511, Turkey; A.Dababat@cgiar.org; 3 Department of Plant Production and Technologies, Faculty of Agricultural Sciences and Technologies, Nigde Omer Halisdemir University, Nigde 51240, Turkey; toktay@yahoo.com; 4 Department of Agricultural Genetic Engineering, Faculty of Agricultural Sciences and Technologies, Nigde Omer Halisdemir University, Nigde 51240, Turkey; abthebest@gmail.com; 5 College of Agriculture, University of Sargodha, Sargodha 40100, Pakistan; azher490@hotmail.com; 6 National Engineering Laboratory for Crop Molecular Breeding, Institute of Crop Sciences, Chinese Academy of Agricultural Sciences, Beijing 100081, China

**Keywords:** cereal cyst nematodes, wheat, barely, breeding, biotechnology, resistance

## Abstract

Cereal cyst nematodes (CCNs) are among the most important nematode pests that limit production of small grain cereals like wheat and barley. These nematodes alone are estimated to reduce production of crops by 10% globally. This necessitates a huge enhancement of nematode resistance in cereal crops against CCNs. Nematode resistance in wheat and barley in combination with higher grain yields has been a preferential research area for cereal nematologists. This usually involved the targeted genetic exploitations through natural means of classical selection breeding of resistant genotypes and finding quantitative trait luci (QTLs) associated with resistance genes. These improvements were based on available genetic diversity among the crop plants. Recently, genome-wide association studies have widely been exploited to associate nematode resistance or susceptibility with particular regions of the genome. Use of biotechnological tools through the application of various transgenic strategies for enhancement of nematode resistance in various crop plants including wheat and barley had also been an important area of research. These modern approaches primarily include the use of gene silencing, exploitation of nematode effector genes, proteinase inhibitors, chemodisruptive peptides and a combination of one or more of these approaches. Furthermore, the perspective genome editing technologies including CRISPR-Cas9 could also be helpful for improving CCN resistance in wheat and barley. The information provided in this review will be helpful to enhance resistance against CCNs and will attract the attention of the scientific community towards this neglected area.

## 1. Introduction

Small grain cereals have served as the basis for staple foods, beverages, and animal feed for thousands of years [1,2,3]. Wheat (*Ttriticum aestivum* L.), barley (*Hordeum vulgare* L.), oats (*Avena sativa* L.), rye (*Secale cereale* L.), triticale (*X Triticicosecale* Wittm.), rice (*Oryza sativa* L.), and some other cereals are rich in calories, proteins, carbohydrates, vitamins, and minerals. These cereals supply around 20% of the calories consumed by people worldwide and are therefore a primary source of energy for humans. Global production of small grains increased exponentially from 1960 to 2005, and then began to decline [4]. Further decline in production is projected to continue through 2050 [5], while global demand for these grains is projected to increase by 1% per annum [4]. 

Currently, wheat, barley, and oat production exceeds consumption in developed countries, while in developing countries the consumption rate is higher than production [5]. Current production levels and trends will not be sufficient to fulfill the projected global demand spawned by the increasing population pressure. For wheat, global production will need to be increased by 60% to fulfill the estimated demand in 2050 [6]. Until recently, global wheat production increased mostly in response to development of improved cultivars and farming practices and technologies. Production is now limited by biotic and abiotic constraints, including diseases, nematodes, insect pests, weeds, and climate. Among these constraints, plant-parasitic nematodes (PPNs) alone are estimated to reduce production of all world crops by 10% [7]. Nematodes are the second biggest group of animals after insects and are present everywhere on the earth ranging from the Polar Regions to the bottom of the oceans [8]. They are present in the ecosystem as free living and as saprophytes, bacteriovores, fungivores, algaevores and as parasites of human, animals and plants. PPNs are 7% of total species of the phylum Nematoda belonging to 4300 species and 197 genera and infect a huge range of economically important crop plants, including wheat and barley [9,10]. The most dangerous species of PPNs belong to Heteroderidae, which also exhibits the cereal cyst nematodes (CCNs). These nematodes are obligate sedentary endo-parasites and are among the important pests that limit the production of small grain cereals. Heavily invaded young cereal plants are stunted and their lower leaves are often chlorotic, forming pale green patches in the field. Mature plants are also stunted and have a reduced number of tillers, and the roots are shallow and abbreviated and have a “bushy-knotted” appearance [11,12]. CCNs comprise a number of closely-related species and are found in most regions where cereals are produced [13,14,15,16,17]. Several efforts have been made to enhance resistance against CCNs in wheat and barley. A major proportion of these efforts are screening and selection of suitable parents for breeding programs followed by use of molecular markers associated with this resistance. Similarly, some attempts have also been made to silence certain nematode parasitism genes and to develop plants with increased resistance using biotechnological approaches. This review is an update on the use of various classical and modern approaches to induce resistance against CCNs in wheat and barley. 

## 2. Economic Impact of CCNs in Wheat and Barely

CCNs have reduced yields in individual research trials or fields by as much as 20% in Pakistan, 50% in Australia, 50% in Turkey, and 90% in Saudi Arabia [14,18]. More than half the fields are reported to be infested by CCNs in selected cereal-producing regions of Turkey [19,20], Iran [21], the U.S.A. [22], and Europe [23]. Wheat fields are infested by CCNs in at least 16 provinces of China [24]. A bibliography of 123 CCN publications relating to all aspects of CCN biology and management in China, from 1991 to 2014, was published by Riley and Qi [25]. Reports of crop losses at the magnitudes shown above do not accurately depict the magnitude of economic losses at the regional or national level because documentation was based mostly on research plots located in infested areas of fields. Since the nematode density varies greatly across most fields, published estimates nearly always fail to represent field-wide yield reductions, which are rarely documented. A further complication is that some reports initially attributed to yield reduction by *H. avenae* are now known or assumed to have been attributable to species recently reclassified as *H. australis*, *H. filipjevi*, *H. latipons*, or *H. sturhani*. Nevertheless, several reports of regional or national crop losses caused by CCNs are available. In Australia, annual yield losses due to Australian populations of *H. avenae* were estimated at 300 thousand tonnes [26]. Losses in Australia were at one time much higher but have been reduced greatly by deploying resistant varieties because only one biotype is present [18,27]. Yield losses in three provinces of China, caused by *H. avenae*, were estimated at 1.2 Mt, assuming that 22% of the production area was infested and that the overall yield reduction was 10% in those areas [24]. National production of cereals in Norway was estimated to be reduced by between 1% and 5% by multiple CCN species [28]. Losses from *H. avenae* and *H. filipjevi* in four northwestern states of the U.S.A. are estimated at 22 thousand tons, assuming that 0.04% of the wheat and barley fields are infested and the average field-wide yield reduction in infested fields is 10% [29]. Economic losses in other infested regions of the western U.S.A. and globally are poorly documented [14,30,31]. 

## 3. Factors Affecting Yield Losses in CCN-Disease

Nematode population has a strong correlation with yield reduction and this relationship is primarily influenced by crop variety, prevailing climatic conditions, cultural techniques, soil quality, and the distribution and density of nematode at a particular location. Similarly, improper use of fertilizer, drought conditions, weakness of root proliferation in soil and unfavorable temperature affected plants are also important determinants of nematode density at the planting time. The varieties have the capability to replace the damaged roots, and thus it is considered to have a better potential among varieties. Nevertheless, associations have been made throughout the history of CCN research to demonstrate a generally linear relationship between initial population density and potential for reduced grain yield, and reductions of other growth and yield components. As an example of the relationship between CCN density and grain yield, *H. filipjevi* in Iran reduced the yield of rainfed winter wheat at all densities ranging from 2.5 to 20 eggs plus J2s/g of soil, with the lowest and highest densities causing 11 and 48% reductions in yield, respectively [32]. Andersen [33] reported that the numbers of *H. avenae* cysts produced on barley were 12, 24, 26, and 33 cysts/plant at initial densities of 1, 2.5, 5, and 10 eggs plus J2s/g of soil. In the northwestern U.S.A., rainfed wheat yields are generally reduced when the number of *H. avenae* eggs plus J2s from extracted cysts, present within the soil, exceeds 3/g of soil [29]. Densities of five *H. avenae* or *H. filipjevi* J2s/g of soil are capable of causing economic damage to irrigated wheat in India [34]. This demonstrates that the initial population pressure is a key player involved in the reduction of grain yield in addition to various other biotic, abiotic and edaphic factors associated with the plants and nematodes.

## 4. Resistance and Tolerance Responses against CCNs 

Use of host-plant resistance and tolerance against CCNs is one of the most cost effective and prominent approaches to minimize crop losses below the threshold level [35]. Continuous cultivation of resistant wheat or barley crops has successfully reduced CCN densities to negligible levels in several countries around the globe (Reviewed by Smiley et al. [29]). Cook and Evans [36] and Cui et al. [37] documented the suppression of nematode reproduction due to the plantation of resistant cultivars. It is established that most of the resistances reported against CCNs in commercial cultivars have been based on introgressions of single dominant genes [12,15,38,39,40,41]. However, the resistance response must be combined with tolerance to attain yield stability [36,42]. Similarly, Cook and Evans [36] reported that the ability to get better crop yield from tolerant cultivars is possible, which is normally coupled with a substantial degree of nematode control. However, generally tolerance is tested under field conditions by comparing the yield of a control treated with a nematocide, i.e., aldicarb, and untreated infested soil that is also used to assess the effect of pre-planting nematode population density [16,42,43].

Roots of resistant and susceptible cultivars are initially attacked by J2s, which may result in an intolerant reaction prior to the expression of resistance in a resistant cultivar [44,45,46,47,48]. The tolerance character is primarily attributed to particular characteristics of root growth and physiological response of the plants to nematode invasion [49,50]. During the course of establishment of syncytia, root development is highly compromised due to root abbreviation and sometimes proliferated adventitious roots. The growth of infected roots, in the increasing depth of the soil, mostly failed. Nematode resistance is mostly negatively associated with grain yield and susceptible cultivars without nematode infestation showed a higher grain yield as compared to that of resistant cultivars [51]. Due to this reason, farmers mostly show unwillingness to use resistant cultivars, which presents a lower yield than susceptible in non-infested soils [23].

Andersson [44] and O’Brien and Fisher [45] concluded that barley normally shows more tolerance against *H. avenae* as compared to oats or wheat. Andersen [33] reported comparative damage thresholds of 5, 1, and 0.2 eggs plus J2s per gram of soil for these crops, respectively. Those results were further confirmed in selected cultivars of barley and wheat in the U.S.A. [43]. The mechanism behind this enhanced tolerance in barley is generally associated with earlier development of crown roots in barley seedlings than in wheat seedlings, which enables the crown roots to compensate more rapidly for early damage on seminal roots [52]. 

On the other hand, a combination of resistance and tolerance responses in wheat and barley cultivars leads to improved profitability and production efficiency [29]. However, most wheat breeding programs in the U.S.A. and other regions with established populations of CCNs are not yet breeding for resistance or selecting for tolerance against CCNs. Less financial support, technical and institutional support, uniformity of infestation and a lack of field testing sites could be the possible reasons behind this. Nonetheless, an understanding of accurate resistance mechanisms would be the key to enhance nematode resistant and/or tolerant wheat and barley cultivars [53,54,55,56]. Various isolates of *Heterodera* from different regions of the world differ in their behavior against the resistance response in different cereal crops. The resistant cultivars of wheat, barley, or oat to the populations of *Heterodera* in one region are susceptible to the CCN populations in the other region [29]. Oat cultivars exhibiting resistance and tolerance to *H. avenae* in Australia were susceptible to *H. avenae* in Britain [57]. 

## 5. Marker-Assisted Breeding and QTL Mapping for Nematode Resistance

The first resistance gene against *H. avenae* was discovered in barley during 1920 in Sweden; however, it was not characterized until 1961 [33]. After that, a number of scientists in several countries with five decades focused on the improvement of resistance in barley against CCN (Reviewed by Smiley et al. [29]). The source of resistant to *H. avenae* was found on chromosome 2H of barley, which was mapped as the *Ha2* locus [58] through restriction fragment length polymorphism (RFLP) molecular markers. Similarly, Barr et al. [59] mapped *Ha4* locus on chromosome 5H in barley using RFLP.

It is well established that most of the resistant sources against various diseases in common wheat were obtained from wild wheat relatives through breeding programs [46]. Barloy et al. [60] reported that 9 resistance genes were transferred into common wheat from its wild relatives like *Aegilops* and other *Triticum* spp. to enhance resistance against *H. avenae*. These genes include *Cre1* and *Cre8* from *T. aestivum*; *Cre3* and *Cre4* from *Ae. tauschii* Coss.; *Cre2*, *Cre5* and *Cre6* from *Ae. ventricosa* (Zhuk.); *Cre7* from *Ae. triuncialis* L.; *CreR* from rye and *CreV* from *Dasypium villosum* L. Can. [61]. Recently, Baloch et al. [62] have reviewed and given detailed information regarding the transfer of these genes and their associated QTL in common wheat from its wild relatives.

Furthermore, some other resistance loci, i.e., *CreX* and *CreY* from *Ae. variabilis* Eig. [60], were documented, while their location on the chromosomes and mode of inheritance is still uncharacterized. Similarly, in the hexaploid wheat, many resistance genes are introgressed from its progenitors. Mokabli et al. [63] and Rivoal et al. [41] reported that *Cre1* gene is highly responsive against *H. avenae* population in north Africa, Europe, and North America, while in Asia and Australia it is less effective or ineffective against the population of CCNs [41,63]. However, the virulence to *Cre1* gene, compared with *H. avenae*, differs for the population of *H. latipons* in Syria and in India for *H. filipjevi* [63]. Gene *Cre1* was effective against *H. filipjevi* but *Cre3* was not effective against the Turkish population of *H. filipjevi* [29]. *Cre3* is mostly effective against the population of *H. avenae* in Australia [64], while not being effective against the European populations of *H. avenae* [65,66]. Nicol et al. [67] reported that *Cre2* and *Cre4* genes from *Aegilops* spp. along with an unidentified gene from wheat line AUS4930 have broad spectrum resistance against several species of genus *Heterodera* and its pathotypes. The coordination of CIMMYT established these loci in wheat in many regions of the world, which included *Cre1* to *Cre7* with a substantial degree of resistance against CCNs. The CCN resistance QTLs are mapped on chromosomes 1A, 1D, 4D, 5A, 5B, 5D, 6A, 6B, 7A, and 7D of hexaploid wheat [68,69]. Additionally, 11 DArT markers associated with resistance against CCNs have also been reported in wheat and could lead to the identification of new resistance loci and tools that may become useful in wheat breeding programs [68]. Smiley et al. [29] reviewed the findings of previous scientists that a single resistance gene to *H. avenae*, which has been used for the identification of new pathotypes for a long period of time in wheat, oat, and barley. 

Some researchers have developed the molecular markers linked to the resistance against *H. avenae* in wheat and barley (Reviewed by Smiley et al. [29]). Marker-assisted selection (MAS) and backcrossing to improve genetic resistance is being applied, but effective resistance genes for CCNs are not yet available for all crops and are not effective against all pathotypes. Large scale tube, pot, or field test screening to identify lines of wheat, barley, oats, and triticale resistant to the Australian populations of *H. avenae* have been undertaken in Australia for more than 30 years [27]. Initially, the pot test was the method of choice, with resistance determined by white cysts counted on the surface of root balls enabling up to 600 pots to be evaluated each day. Up to 130,000 plants per annum have been screened in this way, resulting in the release of many cultivars resistant or moderately resistant to the Australian populations [27]. However, this approach is labor intensive and time consuming, taking a full growing season to complete. With the development and validation of codominant molecular markers linked to resistance to the Australian populations of *H. avenae*, selection can be applied to leaf samples from small seedlings, and the tests can be automated to determine the presence of resistance genes in 1 to 2 days, with substantial savings in costs and time. As a result, MAS for resistance to CCNs in wheat is now used routinely in Australia to identify resistant germplasm in breeding programs [29].

The combination of pot tests and MAS has been used very successfully to reduce infestation levels and losses caused by the CCN in Australia [70]. The strategy followed for MAS involves two phases: prebreeding, to identify and characterize resistant sources and the development of linked markers, followed by their incorporation by backcrossing into advanced breeding lines. This includes pyramiding of resistance genes from different sources (e.g., *Cre1* on chromosome arm 2BL, *Cre3* on chromosome arm 2DL, and *Cre8* on chromosome arm 6BL), using specific linked PCR-based molecular markers to follow each gene [70]. Deployment of resistant cultivars, starting in about 1975, was also responsible for a strong decrease in damage caused by *H. avenae* in Sweden [44].

Transgenic expression of resistance genes results in the induction of a variety of defense responses, including the up-regulation of pathogenesis related (PR) proteins to establish nematode resistance in plants [71]. Uehara et al. [72] reported that induction of PR-1(P4) involved in the regulation of resistance conferred by *HeroA* R gene against potato cyst nematodes [PCNs, *Globodera rostochiensis* (Wollenweber, 1923) and *G. pallida* (Stone, 1973)] through salicylic acid (SA) signaling and nematode infection resulted in the inhibition of the SA signaling pathway in the susceptible cultivars. Similar effects were found in resistant line of hexaploid wheat carrying *Cre2* gene, which showed upregulation of ascorbate peroxidase coding gene in response to cereal cyst nematode (*H. avenae*) when compared with the expression in the susceptible lines [73].

## 6. Genome-Wide Association Studies for CCN Resistance 

Different molecular markers linked to genes for nematode resistance have been identified and reported by developing and utilizing bi-parental populations acquired by crossing contrasting parents, i.e., resistant and susceptible to particular isolates of CCNs. This provides the basis of QTL mapping for nematode resistance which normally involves at least a couple years to develop mapping populations and gene discovery based on the genetic background of only two parental genotypes. Recently, 3 QTL linked to the genomic regions associated with resistance against *Pratylechus thorni* have been identified on chromosomes 2B and 6D in wheat, validating the robustness of these QTL as useful sources of resistance in different genetic backgrounds [74]. Wheat genotypes resistant to *H. avenae* in one region of a same country could be highly susceptible to isolates of *H. avenae* from other regions. This was confirmed by Imren et al. [75] who assessed various landraces and national wheat cultivars from Turkey that wheat genotypes and isolates from different regions in the same country behaved differently. Moreover, QTL recognized from a particular genetic background and working in a particular environment may not be similarly responsive in the other. This shows the need for the development and use of other alternative strategies like association mapping (AM) for the identification of molecular markers which are tightly associated with nematode resistance in crop plants.

Association mapping makes the use of natural plant populations instead of developing the bi-parental mapping populations. However, substantial diversity is required in AM for the detection of QTL with enhanced resolution because AM employs linkage disequilibrium (LD) between alleles within diverse populations to identify markers associated with particular traits. This technique primarily involves genotyping of crop populations, i.e., landraces, cultivars, and advanced lines etc., using particular molecular markers like simple sequence repeats (SSRs) and single-nucleotide polymorphisms (SNPs) and simultaneously phenotying this particular collection of germplasm against a specific biotic stress. For instance, Yang et al. [76] used this approach to map genomic regions associated with stripe rust resistance in wheat. Likewise, recent approaches, such as genome-wide association mapping, utilize the adapted germplasm for identification of QTL associated with a particular plant trait. Moreover, comparing with bi-parental mapping populations, in addition to higher mapping resolution, genome-wide association mapping leads to an increased number of identified alleles and immediate application of the mapping results in a breeding program [62]. 

Recently, genome-wide association studies (GWAS) have been largely exploited to map the genomic locations associated with a specific plant trait. This approach comprises the high-throughput genotyping of germplasm accessions by sequencing (GBS) through PCR based and next generation sequencing technologies followed by the phenotying. This genotyping results in high-density SNP data that could be utilized to detect marker-character associations in the mapping experiments [77,78,79]. Advancement in next-generation sequencing technologies has considerably enabled the discovery of SNPs by whole genome sequencing [80,81,82], transcriptome sequencing [83,84], or reduced-representation sequencing in diverse populations of individuals [85]. GWAS could also be an effective way to identity resistance QTL associated with nematode resistance. For instance, very recently, 13 SNPs associated with resistance to soybean cyst nematode (*Heterodera glycines* Ichinohe, 1952) were recognized, out of which 3 SNPs were associated with already reported QTLs *Rhg1* and *Rhg4* [86].

Similarly, Pariyar et al. [87] performed GWAS of 161 winter wheat accessions using 90K iSelect SNP chip and identified 11 QTL on chromosomes 1AL, 2AS, 2BL, 3AL, 3BL, 4AS, 4AL, 5BL, and 7BL associated with resistance against *H. filipjevi*. They further reported that 8 of these 11 QTL present on chromosome arms 1AL, 2AS, 2BL, 3AL, 4AL, and 5BL were tightly associated with putative genes important for resistant response during plant-pathogen interactions. For instance, methyl transferase 1-associated protein 1 (DMAP1) gene and a putative a RING/FYVE/PHD-type Zinc finger gene linked to chromosome arms 1AL and 2AS are involved in programmed cell death and resistant response against pests. According to the authors, it was the first report of GWAS to map the resistance against CCNs in wheat. 

GWAS was recently used to identify Diversity Array Technology (DArT) markers associated with resistance against *H. avenae*, *P. neglectus*, and *P. thornei* from 126 CIMMYT advanced lines of spring wheat adapted to semi-arid conditions [68]. The results demonstrated association of 11 markers with resistance against Ha21 pathotype of *H. avenae*, 25 markers with resistance against *P. neglectus*, and 9 significant markers linked to resistance against *P. thornei*. In that work, chromosome 4A (~90–105 cM) proved to be a source of resistance to *P. thornei*, as has been recently reported. All the QTL associated with resistance against *H. avenae* were mapped on chromosomes 5A, 6A, and 7A of wheat.

## 7. Nematode Resistance through Modern Approaches

Conventional breeding for pest resistance requires either large scale screening of genotypes to identify suitable resistance genes and multiple generations of plants to incorporate the resistance, or more recently, the application of MAS to combine validated resistance genes. Transgenic plants provide an alternative strategy to develop new forms of resistance by homologous or heterologous transfer of functional resistance genes directly, or by developing ‘synthetic’ resistance genes that interfere with vital processes in the nematode-plant interaction. We have recently reviewed various transgenic strategies to enhance nematode resistance in plants [10]. Here, some of these important approaches are discussed to develop plants that are resistant against CCNs.

### 7.1. Gene Silencing for the Enhancement of Nematode Resistance

PPNs can take up macromolecules from the cytoplasm of plant cell during the feeding process. Initially it was thought that J2s would not ingest external solution when outside a host plant. It is now evident that J2s can take up external solutions containing dsRNA when they are ‘soaked’ or incubated in a solution containing dsRNA [88], with some studies showing that the addition of neurostimulants like octopamine to the soaking solution can enhance the uptake of solution and thus dsRNA by PPNs. This finding enabled the study of gene silencing through in vitro soaking or feeding of dsRNA to the infective J2s. As a result, many effectors and other genes involved in biochemical and developmental processes have been targeted in functional studies to determine how their silencing can affect nematode parasitism, reproduction, viability and ability to establish feeding sites [10]. In general, in vitro studies involving the soaking of J2s in dsRNA solutions for up to 24 h have been used to assess the phenotypic effects (e.g., reduced motility or rigidity, aberrant behavior, reduced attraction to roots, reduction in migration in roots), and subsequent quantification of changes in the expression of the targeted gene [89,90]. Such studies target genes involved in nematode parasitism, development, locomotion, invasion and important biological pathways. To date, there have been relatively few studies on gene silencing for CCNs, partly because these species are relatively difficult to handle and culture, and because genomic or transcriptomic data were rather limited. Recently, Gantasala et al. [91] used in vitro silencing of 4 genes from *H. avenae* viz., nuclear hormone receptor, ployadenalyte binding protein, intron binding protein and epsin, through siRNA soaking. They reported that silencing of these genes resulted in a 71%, 26%, and 60% reduction in females and eggs due to the silencing of epsin, intron binding protein and ployadenalyte binding protein, respectively. Conversely, a 25% increase in females and eggs was reported due to the suppression of the nuclear hormone receptor [91].

**Figure 1:**
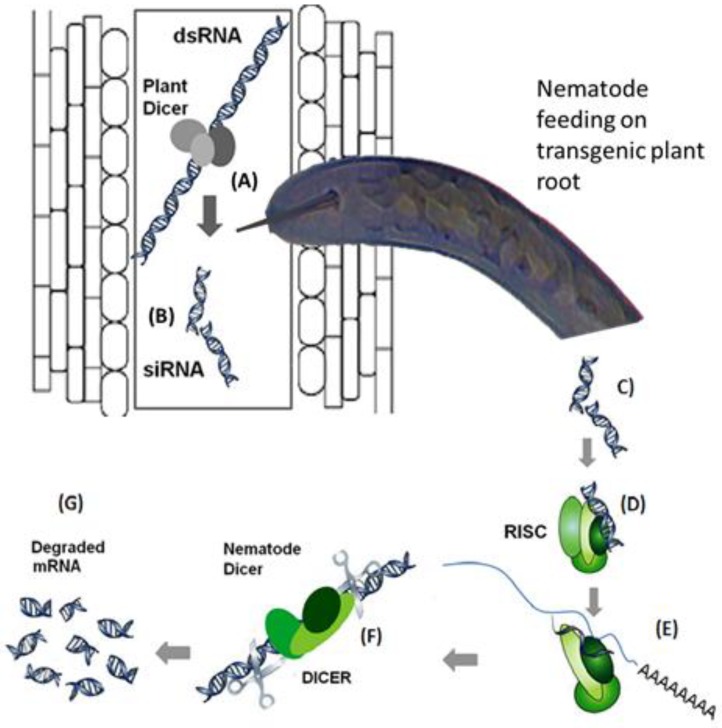
Host induced gene silencing for nematode resistance in plants. (**A**) and (**B**): Double stranded RNA (dsRNA) sequence of the target nematode effector gene is transformed into the plant which is cut by the plant DICER enzyme into small interfering RNAs (siRNAs). (**C**), (**D**) and (**E**): These siRNAs are taken up by the nematodes through their stylets which are detected by the RISC complex that binds to mRNA (complementary to the siRNA sequences) of the target effector gene. (**F**) and (**G**): This is followed by the activation of nematode DICER to cut the double stranded RNA to degrade the mRNA of the target nematode effector gene. Reproduced from Ali et al. [10].

Host-induced gene silencing (HIGS) is another transgenic approach that has been widely used by the scientists to silence nematode genes involved in the invasion, virulence and establishment of feeding sites. This technique involves the cloning and transformation of dsRNA of nematode gene into the host plants from where it is taken up by the nematodes through their sylet in the form of siRNAs. These siRNAs interfere with expression of nematode genes at the transcript level to induce silencing of that particular gene. A diagrammatic representation of HIGS in provided in Figure 1. We have recently reviewed gene silencing studies that have been undertaken for cyst nematodes [10,29,92].

In most of the HIGS studies, different goals set by the researchers have been achieved by knocking down the vital genes from nematodes to reduce their ability to parasitize the plant, or by interfering with chemotaxis towards and invasion into host roots, migration into the root tissues, development of nematode feeding structures, or reproduction (reviewed by references [8,10,93]). For example, some data indicates that more than a 90% reduction can be achieved in cyst development for *H. schachtii* on *Arabidopsis*, and a reduction in cyst formation of up to 94% was found after silencing the synaptobrevin (*snb-1*) gene in soybean cyst nematode through HIGS [94]. It is important to perform bioinformatic analyses of the target gene sequences before using them in the gene silencing studies to get rid of off-target effects. In addition, this approach can be used to silence genes that are induced in the host plants and interact with nematode-secreted effector proteins for compatible plant-nematode interactions. For example, the CLE-like nematode effectors are recognized by plant CLE receptors that are required for syncytium development in plants [95,96]. Knockdown of these receptors in soybean roots resulted in reduced soybean cyst nematode infestations [97]. However, reducing expression of plant genes involved in the nematode-plant interaction is likely to be detrimental to plant crops, since the plant genes will have a functional role in the plant, and interfering with this could reduce field performance. To overcome this problem, we have recently reported the site specific delivery of nematode resistance genes and miRNAs of plant genes involved in the establishment of nematode feeding sites using syncytia specific promoters [98,99].

Some of the nematode effector proteins are localized in the nucleus of the host plant cells, which act as transcription factors to regulate the expression of plant genes [92]. It has been shown recently that annexin like protein (Ha-annexin) from *H. avenae* is localized in the nuclei of host plant cells and is involved in the suppression of basal plant defense responses [100]. A transgenic wheat line containing a HIGS construct of Ha-annexin revealed compromised nematode establishment on the plants. Similarly, the transient expression of Ha-annexin led to the down-regulation of the host hypersensitive (HR) response induced by BAX protein and different pathogen associated molecular patterns (PAMPs) such as flagellin [100]. Very recently two venom allergen-like effector proteins (HaVAP1 and HaVAP2) from *H. avenae* have been characterized through gene silencing [101]. The results indicated that both of them are involved in the suppression of programmed cell death induced by BAX in *Nicotiana benthamiana* leaves. Kumar et al. [102] have reported the de novo transcriptome of *H. avenae* which may lead to the identification of various candidate target genes for HIGS to enhance CCN resistance in wheat. 

However, most of the times, HIGS using RNAi does not confer 100% resistance to nematodes. There are a number of possible explanations for this. For instance, the first reason could be the specific target gene chosen with several questions like, is its expression vital, is it unique, or does it belong to a multigene family? Similarly, other factors that affect the extent of gene silencing include where and when the target gene is expressed, the specific dsRNA sequence chosen, the stability of target mRNA or encoded protein, the presence of a ‘recovery’ phenomenon, and experimental variables such as the vigor of the nematodes treated, and differences in the RNAi machinery (e.g., for systemic spread of the silencing signal) between genera or species [103]. This shows the need for other appropriate biotechnologies to increase nematode resistance in plants.

### 7.2. Utilization of Proteinase Inhibitors and Chemosensory Disruptive Peptides 

RNAi is not the only transgenic approach to confer resistance to nematode pests. Quite a number of different protease inhibitors (PIs) such as cystatins, cowpea trypsin inhibitor (CpTI) and serine proteinase inhibitors have been documented to be successful in producing nematode resistant plants [104]. Heterologous expression of a serine proteinase inhibitor, PIN2 from potato (*Solanum tuberosum* L.) into durum wheat (*Triticum durum* Desf.) led to increased resistance in wheat against *H. avenae* [105]. The main digestive enzymes of many nematodes are cysteine proteinases which have been tackled with the transgenic expression of small proteinase inhibitors, i.e., cystatins, in several plants species to enhance nematode resistance. This strategy has been successfully utilized in a number of crop plants like tomato (*Lycopersicon esculentum* Mill.), rice (*Oryza sativa* L.), potato, banana (*Musa saplentum* L.), and plantain [10]. The human diet normal part is cystatin and is quickly sullied by gastric juice and also it is not allergenic, indicating there is no biosafety issue: the peptide is too small to be allergenic and is degraded in the human small intestine. Similarly, there is no evidence for environmental safety concerns. 

In addition to utilization of anti-feedant cysteine proteinase inhibitors to interfere with nematode digestion, anti-root-invasion non-lethal synthetic peptides have been used to hinder the invasion of the nematodes in host plants. These peptides are able to disrupt the chemosensory ability of the nematodes, which make them unable to sense the presence of the plants and the process of nematode chemotaxis towards plants is greatly affected. Winter et al. [106] first reported that synthetic peptides can interfere with the chemoreceptive ability of nematodes to chemically signal in very small concentrations. It was reported that expression of a synthetic peptide nAChRbp in potato resulted in the inhibition of nematode acetylcholinesterase (*AChE*) gene, leading to disorientation of invading J2s of PCN (*G. pallida*) that led to a 52% reduction of female nematodes established on potato roots [107]. Moreover, Costa et al. [108] revealed that *AChE* is highly expressed in chemo- and mechano-sensory neurons of *C. elegan,* further supporting the hypothesis that inhibition of this gene results in chemodisrution of the invading J2s of PPNs [109]. These reports suggest a potential use of chemoreceptive nematode repellent peptides to induce transgenic resistance in cereal crops against various species of CCNs.

### 7.3. Coupling of Various Resistance Strategies to Augment Nematode Resistance

Sometimes, if the HIGS does not work efficiently, it could be coupled with some other transgenic approaches to augment nematode resistance in plants. One way to improve transgenic resistance to nematodes is to combine two different modes of resistance, such as a cystatin and anti-invasion peptide [110]. The coupling of these approaches in potato has resulted in high degree of resistance against PCN without effecting soil quality [111]. The same combination approach has been utilized by the scientists at International Institute of Tropical Agriculture (IITA, Ibadan, Nigeria), in collaboration with researchers at University of Leeds, UK using maize (*Zea mays* L.) cystatin and synthetic nematode repellent peptide, nAChRbp to developed transgenic plantain for resistance against three nematode species, i.e., *R. similis*, *H. multicinctus* and *Meloidogyne* spp. [112,113]. Furthermore, pyramiding of cystatins and chemodisruptive peptide into different crop plants has shown a high degree of nematode resistance and enhanced crop yields [114,115,116]. These approaches could be further combined with RNAi to give more effective and more durable transgenic resistance. Durability of resistance to pests and diseases is an important consideration, and where RNAi-based traits have already been deployed commercially, the expression of the trait seems to be consistent in the following generations [117,118]. This emphasizes the potential of combining all these approaches to multiply the degree of resistance in cereal crops against various CCN species.

## 8. Genome Editing Technologies: A Potential Perspective for Nematode Resistance in Plants

To date, conventional breeding practices, due to their laborious and time consuming nature, are being replaced with genome editing and other advanced molecular techniques. However, genome editing is being integrated with plant breeding for the development of crop cultivars with improved resistance against pests and other diseases. Until now, different genome editing technologies have been practiced to enhance disease resistance in different crop plants. However, one of the recent and breakthrough in genome editing has been accomplished by the CRISPR (Clustered Regular Interspaced Palindromic Repeats) Cas9 (CRISPR-associated protein) system that is the key player of bacterial immune system.

CRISPR/Cas9 is RNA-guided machinery for effective and precise editing of genomes as compared to any other gene editing technique like Transcription Activator Like Effector Nucleases (TALENS) and Zinc Finger Nucleases (ZFNs) [119,120]. Being precise, highly specific, a multi-gene editor and highly efficient, CRISPR/Cas9 is regarded as promising gene editing system for crop plants [119]. Because CRISPR/Cas9 technology is sequence specific nuclease, it could be employed to exploit defense related mechanisms in plants against invading pathogens. For instance, Ali et al. [119] and Baltes et al. [120] described the implementation of the CRISPR/Cas9 technique to develop resistant plants against geminiviruses and exhibit great potential for enhancement of CCN resistance in cereal crops. 

Genome editing using this system is an exciting and powerful alternative to RNAi for gene silencing. It is essentially targeted mutagenesis, in which mutations can be induced in target genes in a nontransgenic manner termed nonhomologous end joining (NHEJ). Alternatively, by insertion of oligonucleotide sequences with ends homologous to each side of the cut site, specific additions to a sequence can be made, known as homologous end joining (HEJ). The technology has passed through a number of iterations, with the use of a ‘guide’ RNA sequence directing a dsDNAse enzyme (CRISPR-Cas9) to cut a target sequence at a specific site [121]. In NHEJ, the cell repair enzymes frequently make a mistake in joining the ends, resulting in a targeted mutation or total inactivation of the gene. When a cassette consisting of a selectable marker gene with CRISPR and Cas 9 is used to select edited cells for regeneration to plants, the site of gene editing will be elsewhere in the genome from the editing cassette. Hence, for cereals, it is possible to generate edited genotypes that no longer contain an editing cassette. This can be achieved by making a cross with the original (or another) genotype, and identifying genotypes with the edited gene but without the introduced cassette. A plant with a targeted mutation but lacking any introduced DNA may well be regulated as non-Genetically Modified (Non-GM) [29]. However, since the silencing trigger is delivered from the plant to the pest in HIGS, it is not possible to deliver a (nontransgenic) genome editing signal in this way. Application of this technology for nematode management necessitates the identification of a nonvital host plant gene whose expression is very much needed for nematode parasitism to edit it to be nonfunctional. 

Recently, genome editing using CRISPR/Cas 9 system has been established in free living nematode, *Caenorhabditis elegans* [122,123]. This development would lead to the characterization of several important genes involved in different physiological processes of nematodes. Nonetheless, there are very few reports available on the application of CRISPR/Cas 9 system to study the resistance responses of the plants against nematodes. Kang [124] recently used soybean hairy roots to study the resistance response knockouts of two serine hydroxymethyltransferase genes, *GmSHMT08* and *GmSHMT05*, generated through this system in soybean-*H. glycines* model organisms. However, to date no report is available regarding the use of this technology in CCN-plant interactions. The recent availability of genome sequences for hosts of CCNs could well lead to the development of nontransgenic cereals genome edited for resistance [125,126].

## 9. Conclusions and Future Perspectives 

Current production levels and trends of wheat and barley will not be sufficient to fulfill the projected global demand created by increased populations. For wheat, global production will need to be increased by 60% to fulfill the estimated demand in 2050. Recently, the global wheat production has increased mostly in response to the development of improved cultivars and farming practices and technologies. However, its production is still limited by biotic and abiotic constraints, including diseases, nematodes, insect pests, weeds, and changing climate. Since domestication, improvements in crop plants regarding pest resistance and higher yields are preferential research area of all times in plant sciences, and usually involve targeted genetic exploitations through natural means as well as transgenic means.

Genotyping by sequencing (GBS) followed by association mapping of resistance genes in crop plants could be an important strategy. Similarly, whole genome sequence availability of various CCNs could provide a great platform regarding the vital effector genes that could be manipulated via different transgenic technologies [126,127]. Moreover, full sequence and annotation of wheat genome has been carried out recently containing huge data that could be helpful in understanding nematode-wheat interactions at the molecular level, which could be used for the enhancement of resistance [127]. Furthermore, wheat and barley transcriptome studies in response to CCN infection could be used to manipulate different up- and down-regulated genes. The genes involved in nematode establishment could be down-regulated and suppressed defense genes in syncytia could be overexpressed using syncytia specific genes, as has been demonstrated in Arabidopsis [10,97,98]. Recently, the role of silicon derived resistance has been described in plants against a variety of phytopathogens [128]. This silicon based resistance could be employed for managing CCNs.

Although about 10% of the world’s crops are transgenic at present, the costs and issues that must be overcome to deploy any form of transgenic crop resistance to nematodes, and to CCN in particular, are not insubstantial. Hence, there is current research aimed at delivering dsRNA in spray form (ectopic delivery) rather than by transgenic plants. This strategy requires low cost production of dsRNA sequences, methods to stabilize them for field delivery, uptake of dsRNA by leaves, its systemic basipetal movement through plants to roots, and uptake by nematode on feeding [129,130]. If the technical aspects of ectopic delivery of dsRNA can be overcome in a cost-effective manner, this could bypass the issues of RNAi-based transgenic nematode control. In addition to ectopic delivery of RNAi, gene silencing technology can be used to determine targets for new nematicides. This process involves genome-enabled novel chemical nematicides [129]. Very recently, it has been reported that the tryptophan decarboxylase 1 (*AeVTDC1*) gene from a wide relative of wheat *Aegilops variabilis* regulates the resistance against *H. avenae* by altering the downstream secondary metabolite contents rather than auxin synthesis [131]. This shows the potential use of genetic resources from wide relatives for the enhancement of CCN resistance in cereal crops.
